# Differences in pain, function and coping in Multidimensional Pain Inventory subgroups of chronic back pain: a one-group pretest-posttest study

**DOI:** 10.1186/1471-2474-12-145

**Published:** 2011-06-30

**Authors:** Martin L Verra, Felix Angst, J Bart Staal, Roberto Brioschi, Susanne Lehmann, André Aeschlimann, Rob A de Bie

**Affiliations:** 1 Physiotherapy Institute, Bern University Hospital, 3010 Bern, Switzerland; 2 Department of Epidemiology, Maastricht University, 6200 MD Maastricht, The Netherlands; 3 Caphri School for Public Health and Primary Care, Maastricht University, 6200 MD Maastricht, The Netherlands; 4 Rehabilitation Clinic "RehaClinic", 5330 Bad Zurzach, Switzerland; 5 Scientific Institute for Quality of Healthcare, Radboud University Medical Centre, 6500 HB Nijmegen, The Netherlands

## Abstract

**Background:**

Patients with non-specific back pain are not a homogeneous group but heterogeneous with regard to their bio-psycho-social impairments. This study examined a sample of 173 highly disabled patients with chronic back pain to find out how the three subgroups based on the Multidimensional Pain Inventory (MPI) differed in their response to an inpatient pain management program.

**Methods:**

Subgroup classification was conducted by cluster analysis using MPI subscale scores at entry into the program. At program entry and at discharge after four weeks, participants completed the MPI, the MOS Short Form-36 (SF-36), the Hospital Anxiety and Depression Scale (HADS), and the Coping Strategies Questionnaire (CSQ). Pairwise analyses of the score changes of the mentioned outcomes of the three MPI subgroups were performed using the Mann-Whitney-U-test for significance.

**Results:**

Cluster analysis identified three MPI subgroups in this highly disabled sample: a dysfunctional, interpersonally distressed and an adaptive copers subgroup. The dysfunctional subgroup (29% of the sample) showed the highest level of depression in SF-36 mental health (33.4 ± 13.9), the interpersonally distressed subgroup (35% of the sample) a modest level of depression (46.8 ± 20.4), and the adaptive copers subgroup (32% of the sample) the lowest level of depression (57.8 ± 19.1). Significant differences in pain reduction and improvement of mental health and coping were observed across the three MPI subgroups, i.e. the effect sizes for MPI pain reduction were: 0.84 (0.44 - 1.24) for the dysfunctional subgroup, 1.22 (0.86 - 1.58) for the adaptive copers subgroup, and 0.53 (0.24 - 0.81) for the interpersonally distressed subgroup (p = 0.006 for pairwise comparison). Significant score changes between subgroups concerning activities and physical functioning could not be identified.

**Conclusions:**

MPI subgroup classification showed significant differences in score changes for pain, mental health and coping. These findings underscore the importance of assessing individual differences to understand how patients adjust to chronic back pain.

## Background

For more than twenty years, a simple diagnostic triage for back pain has been widely accepted and advocated as part of various clinical guidelines to distinguish between possible serious spinal pathology, nerve root pain or simple non-specific back pain [1,2]. By far the largest proportion of the three categories is the non-specific back pain category. Patients with non-specific back pain, according to this diagnostic model, are viewed as a homogeneous group [1]. Various recent randomized controlled trials that have studied the effect of physiotherapy on non-specific back pain, for example, found only small improvements in pain and disability [3,4].

However, both health care professionals and researchers often state that patients with non-specific back pain are heterogeneous with regard to their bio-psycho-social impairments and responsiveness to interventions. Classification of people with chronic "non-specific" back pain into homogeneous subgroups might be an important objective in order to tailor interventions and to control for subgroup differences when evaluating treatment outcome. Consequently, to achieve better clinical outcomes, scientifically confirmed criteria for building subgroups that relate to both diagnoses, pain coping strategies and prognosis of chronic pain patients are required. Thus, it would be possible to fit the treatment modalities to the patient, define the main therapeutic focus and optimally allocate human and financial resources [5-7].

Some randomized trials have demonstrated that effect sizes increase when classification and matching are taken into account [8,9]. However, there are several problems with a sub-grouping approach and it is important that the sub-grouping paradigm is closely examined [10,11]. Several studies have proposed methods for sub-grouping patients with back pain as a means of determining the treatment most likely to benefit patients with particular characteristics, to aid in prognosis or to identify pathology. However, overall consensus has not yet been achieved [12-16]. In their systematic review Billis et al. identified classification systems of low back pain from nine countries [17]. Most studies were classified according to pathoanatomical and/or clinical features, whereas fewer studies utilized a psychosocial and even less, a bio-psycho-social approach. According to the International Classification of Functioning, disability and health (ICF) and the Initiative on Methods, Measurement, and Pain Assessment in Clinical Trials (IMMPACT recommendations), chronic non-specific back pain disorders should be evaluated within a bio-psycho-social framework [18,19].

The Multidimensional Pain Inventory - which measures pain, a number of psychosocial and behavioral variables, and activities - has been used to classify patients with chronic back pain into empirically derived subgroups according to their pattern of symptoms [6,20-22]. In the studies cited above, the Multidimensional Pain Inventory differentiated between three subgroups labelled as adaptive copers, dysfunctional, and interpersonally distressed [20]. The interpersonally distressed cluster is mainly characterized by lower levels of perceived solicitous and distraction responses from the patients' partners or spouses and higher levels of punishing responses compared to the adaptive copers and dysfunctional clusters. The adaptive copers cluster, compared with the other two subgroups, is characterized by less pain severity, less interference with everyday life due to pain and less affective distress, more perception of life control and higher activity level. The persons of the dysfunctional cluster report high pain severity, high interference and activity distress, low life control and low activity level.

The main aim of this study was to examine the score changes on outcomes for pain, physical and mental function, and coping across the Multidimensional Pain Inventory subgroups after a standardized four-week interdisciplinary in-patient pain management program. Based on our previous study on patients with fibromyalgia, we hypothesized that the cluster of dysfunctionals would report, on average, higher improvements than the adaptive copers and the interpersonally distressed in pain, physical function, mental health, and coping [23].

Secondary aims were to investigate whether it was possible to describe the three Multidimensional Pain Inventory cluster solution in line with Turk and Rudy [20-22] and validate the three-cluster solution by comparing the three clusters with measures of pain, psychosocial function, physical and mental health, and pain coping strategies. The hypothesis was that the symptom patterns on the Multidimensional Pain Inventory of the subgroups were similar to those of the corresponding subscales of the Medical Outcome Study Short Form-36, the Hospital Anxiety and Depression Scale, and the Coping Strategies Questionnaire. For example, the dysfunctional were expected to report more pain, worse physical function, and worse mental health than the other two subgroups in the corresponding Short Form-36 subscales.

## Methods

### Setting, participants, and procedure

The study was conducted at the rehabilitation clinic "RehaClinic" (locations Bad Zurzach and Braunwald, Switzerland), which is attended by severely disabled patients suffering from persistent musculoskeletal pain. All subjects were consecutively admitted and included in the study and 1) suffered from chronic non specific back pain (i.e. lumbar, thoracic, or pan vertebral pain syndrome without serious spinal pathology or nerve root pain) and had had pain for at least six months. Further inclusion criteria were 2) ability to complete self-assessment questionnaires, understand the German language, no psycho-intellectual inabilities; and 3) provision of written, signed informed consent. Exclusion criteria were 1) severe somatic illness requiring specific treatment such as cancer, inflammatory rheumatic disease, neurological disease, and pain after a recent operation 2) manifest psychiatric disorder such as dementia, psychosis, suicidality, and 3) failed inclusion criteria.

The patients with chronic non-specific back pain were participants in the "Zurzach Interdisciplinary Pain (German: *Schmerz*) Program" - ZISP. The program is a four-week in-house, standardized, interdisciplinary pain management program. This program has three main components: 1) medical care including adapted drug therapy, 2) exercise therapy and 3) psychotherapy, mainly cognitive and operant behavioral therapy (in total over 100 hours of therapy). The program is intensive. Over the course of treatment patients received on average six daily sessions of the following treatments: physiotherapy, aerobic endurance training, qigong/tai chi exercises, individual psychotherapy including cognitive behavioral therapy, participation in a pain coping group, relaxation therapy, humour therapy, information and education about the pathophysiology of pain mechanisms and management of chronic disabling pain, nursing care, and regular medical consultations including drug therapy. The involved health care providers are: rheumatologists, clinical psychologists, physiotherapists, occupational therapists, nurses, a movement analyst and a humour therapist. Individual treatment strategies were identified and discussed during the interdisciplinary meetings of the pain management team (2 hours per week for six patients). Detailed information on inclusion/exclusion criteria and interdisciplinary treatment goals are published elsewhere [24].

The study protocol was approved by the Local Ethic Commission (Health Department in Aarau, Switzerland, no. EK AG 2008/026). All participants gave written informed consent according to the Declaration of Helsinki.

### Outcome measures

Sociodemographic data were collected using a standardized questionnaire from a previous study [25].

The West Haven-Yale Multidimensional Pain Inventory assesses pain and its consequences in terms of symptoms, disability, activity, behavior, mood, and social relationships [26]. The German version of the Multidimensional Pain Inventory is a self-reported 51-item inventory with eleven subscales: pain severity, interference due to pain, life control, affective distress (synonymously described as negative mood), support, punishing responses, solicitous responses, distracting responses, social and recreational activities, household chores, and activities away from home [27]. The last three subscales can be summarized into one subscale of general activities. The Multidimensional Pain Inventory has been validated in various settings [26-28]. The Multidimensional Pain Inventory has been used to identify subgroups of people with chronic musculoskeletal pain in different settings and with different biomedical diagnoses [28]. Two studies examined the cluster stability of the empirically derived subgroup classification based on the Multidimensional Pain Inventory and found that retest resulted in 65 to 70% of patients being assigned to the same cluster [29,30].

For external validation and calculation of effect sizes, the scales of the instruments listed below were used. The Medical Outcome Study Short Form-36 is a self-administered generic instrument that assesses health-related quality of life [31]. Two physical scales (physical functioning and bodily pain) and two mental scales (vitality and mental health) were examined. We used the validated German version of the Short Form-36 to enquire about symptoms and functioning during the preceding four weeks [32]. The Short Form-36 is used and has been validated in a large number of studies all over the world. The Hospital Anxiety and Depression Scale is a short self-rating of anxiety and depression (seven items each), these being two of the most important affective health dimensions for people with chronic musculoskeletal pain [33]. It has especially been developed and validated in non-psychiatric settings [33,34]. We used the validated German version of the Hospital Anxiety and Depression Scale [34]. The Coping Strategies Questionnaire is a self-report instrument designed to assess the active and passive coping strategies of individuals with chronic pain [35]. For this study we used scales that measure cognitive (catastrophizing) and behavioral coping strategies (increasing activity level). We also used the two one-item scales that assess participants' subjective ability to control or decrease their pain. These two scales measure the perceived effectiveness of participants' coping strategies. We used the validated German version of the Coping Strategies Questionnaire which is a translation and cross-cultural adaptation of the original questionnaire [36]. Validation studies were performed in chronic musculoskeletal pain, including low back pain, and osteoarthritis [35,36].

### Statistical analysis

The outcome measures were assessed on entry to the clinic (pre-treatment baseline) and at discharge after four weeks (post-treatment). The baseline scores of the scales of the Multidimensional Pain Inventory were used for subgroup classification. Differences between the baseline scores of the Short Form-36-/Hospital Anxiety and Depression Scale-/Coping Strategies Questionnaire-subscales of the three Multidimensional Pain Inventory subgroups - as a clue for construct validity - were tested.

The scores of the subscales of the Short Form-36, the Hospital Anxiety and Depression Scale, the Coping Strategies Questionnaire, the Multidimensional Pain Inventory life control/support/solicitous responses/general activities were scaled from 0 = maximal pain/maximal disability/maximal symptoms/worst coping to 100 = no pain/full function/no symptoms/best coping based on a procedure originally described in the Medical Outcomes Study Short Form-36 manual. This scaling was done to facilitate comparison of the clusters on these external validation measures. According to the subgroup classification of Turk and colleagues, the Multidimensional Pain Inventory pain severity/interference with pain/affective distress/punishing responses were scaled from 0 = best to 100 = worst [20].

According to Turk and colleagues, the empirically derived subgroups were defined by confirmatory cluster analysis using a predefined three cluster solution [20]. The Multidimensional Pain Inventory score patterns were depicted as graphs of the mean Multidimensional Pain Inventory baseline scores and compared to the patterns described by Turk and colleagues using the rank orders of the three subgroups within one Multidimensional Pain Inventory subscale. To assess whether a three-cluster solution was appropriate according to mathematical criteria, hierarchical cluster analysis according to the Ward method was performed [37]. Thus, the proposed "best feasible", i.e. clinically characterized, empirically determined solution according to the three Multidimensional Pain Inventory subgroups originally defined by Turk and colleagues was chosen [20].

Effect sizes of the subscales of all four questionnaires were determined by the score difference between entry (baseline) and discharge from the pain management program after four weeks divided by the group standard deviation at entry [38]. Positive effect sizes indicate improvement of the pain condition, while negative effect sizes indicate worsening. An effect size ≥0.80 is considered as large, 0.50-0.79 as moderate, 0.20-0.49 as small, and 0.00-0.19 as very small [38].

Pairwise analyses of the outcomes (entry to discharge) of the three Multidimensional Pain Inventory subgroups were tested using the Mann-Whitney-U-test for significance. In multiple pairwise testing of nonindependent subscale scores (e.g. within the patient rating of pain), the significance level must be reduced by the number of tested subscale scores (k), i.e. p = 0.050/(k![k - 2]! × 2!), which is well known as the Bonferroni correction [39]. Thus, the significance level for the Type 1 error was p = 0.050/6 = 0.008 for the comparisons of the k = 4 scores, p = 0.050/3 = 0.017 for the comparisons of the k = 3, and p = 0.050 for those of two subscale scores.

All analyses were performed using the statistical software package SPSS 17.0 for Windows^® ^(SPSS Inc., Chicago, IL, USA).

## Results

### Participants at baseline

Table 1 describes the demographic and medical data of the total sample of patients with chronic back pain on entry into the pain management program (*n *= 173). The subjects were characterized by a relatively young age, high prevalence and high level of depression, high level of unemployment, and a long history of pain.

**Table 1:** Demographic and medical data for the total sample at entry to the pain management program (n = 173)

Age (years)	
Mean (SD)	46.9 (12.8)
Range	21.3 - 79.6
Sex (%)	
Female	77.8

Marital status (%)	
Single	20.1
Married	72.0
Other	7.9

Education (%)	
Grade 10-12	30.9
High school graduate	50.9
College graduate	14.5
University graduate	3.7

Employment status	
Full time	9.6
Part time	40.1
Unemployed	41.9
Retired	3.6

Mental health (%)	
Depression*	54.5

Pain duration (%)	
7 - 12 months	6.2
13 - 24 months	12.7
25 - 36 months	14.8
37 - 48 months	4.9
49 - 60 months	22.8
>5 years	38.6

SD = standard deviation. *HADS depression cut-off score: ≤9 out of 21 points.

### Classification of persons with chronic back pain into the Multidimensional Pain Inventory subgroups

Table 2 shows the mean baseline scores of the Multidimensional Pain Inventory. One hundred and sixty-seven out of one hundred and seventy-three patients with chronic back pain could be allocated to one of the three chronic pain subgroups. Thirty-five percent (*n *= 61) were classified as "Interpersonally Distressed", 32% (*n *= 56) were classified as "Adaptive Copers", and 29% (*n *= 50) were classified as "Dysfunctional". The six remaining persons with chronic back pain (4%) were classified as "Anomalous" (did not fit into any of the three profiles). The Multidimensional Pain Inventory baseline scores differed between the three clusters: the patients in the dysfunctional cluster showed highest scores for pain severity, interference due to pain, and affective distress and lowest scores for life control and general activities. The cluster interpersonally distressed showed lowest scores for solicitous and distracting responses by their partner or spouses, and the highest score for negative/punishing responses by their partner or spouses. Compared to the other two subgroups, the adaptive copers showed best scores for life control, affective distress, and general activities.

**Table 2:** Mean Multidimensional Pain Inventory subscale baseline scores

MPI subscales	Interpersonally distressed n = 61 (SD)	Adaptive copers n = 56 (SD)	Dysfunctional n = 50 (SD)
Pain severity (6 = worst, most)	4.22 (0.98)	4.64 (0.79)	5.06 (0.71)

Interference with pain (6 = worst, most)	3.89 (0.85)	3.97 (0.88)	5.12 (0.61)

Life control (6 = best, most)	2.89 (1.09)	3.80 (0.82)	1.91 (1.04)

Affective distress (6 = worst, most)	3.33 (1.31)	2.38 (1.11)	4.57 (0.96)

Support (6 = best, most)	2.47 (1.45)	5.35 (0.72)	5.23 (0.88)

Punishing responses (6 = worst, most)	1.75 (1.55)	0.94 (0.96)	1.40 (1.45)

Solicitous responses (6 = best, most)	1.95 (1.05)	4.66 (0.98)	4.42 (1.06)

Distracting Responses (6 = best, most)	2.25 (1.26)	4.10 (1.20)	4.18 (1.36)

General activities (6 = best, most)	2.46 (0.75)	2.48 (0.77)	1.92 (0.86)

MPI = Multidimensional Pain Inventory; n = number of patients; SD = standard deviation.

### Validation of the subgroup classification

Comparing the results of Table 2 and Table 3, the scores of the subgroups of the Short Form-36, Hospital Anxiety and Depression Scale, and Coping Strategies Questionnaire showed almost the same pattern as the Multidimensional Pain Inventory. On the Multidimensional Pain Inventory pain severity, the rank order of the three cluster subgroups in terms of reported pain was dysfunctional (most pain), adaptive copers, and interpersonally distressed (least pain). This same order was found on the Short Form-36 bodily pain: the cluster dysfunctional showed most pain, 14.0 (12.8). The Multidimensional Pain Inventory general activities scale was compared to Short Form-36 physical functioning: the dysfunctional patients showed the lowest activity level (Short Form-36 physical functioning: 34.7 (19.6)). The mean baseline score of Multidimensional Pain Inventory life control was compared to the score of Coping Strategies Questionnaire ability to control pain: the adaptive copers had best control, 48.5 (22.1). Multidimensional Pain Inventory affective distress was compared to Short Form-36 mental health, Hospital Anxiety and Depression Scale depression and anxiety, and Coping Strategies Questionnaire catastrophizing: the cluster dysfunctional showed most and the adaptive copers least affective symptoms on all scales (significant differences in all subgroup comparisons). It was not possible to replicate the specific Multidimensional Pain Inventory characteristics of the interpersonally distressed subgroup (punishing responses and lack of support and distraction of pain by partner or spouse) because the scores of the subscales of the Short Form-36, Hospital Anxiety and Depression Scale and Coping Strategies Questionnaire do not assess social support.

**Table 3:** Mean baseline scores for the three Multidimensional Pain Inventory cluster groups

Subscales	Interpersonally distressed n = 61 (SD)	Adaptive copers n = 56 (SD)	Dysfunctional n = 50 (SD)
SF-36 (100 = best)			
Physical functioning	46.0 (20.7)	43.1 (22.2)	34.7 (19.6)
Bodily pain	23.5 (12.7)	19.5 (11.1)	14.0 (12.8)
Vitality	28.2 (16.6)	35.7 (14.6)	18.2 (15.3)
Mental health	46.8 (20.4)	57.8 (19.1)	33.4 (13.9)

HADS (100 = no anxiety or depression)			
Anxiety	52.5 (22.1)	60.5 (18.5)	37.5 (15.4)
Depression	57.5 (20.1)	65.1 (18.4)	39.9 (18.3)

CSQ (100 = best)			
Catastrophizing	50.2 (17.9)	51.2 (15.4)	35.1 (17.2)
Increasing activity level	55.6 (15.0)	63.8 (13.4)	51.5 (16.9)
Ability to control pain	48.0 (24.4)	48.5 (22.1)	33.3 (22.8)
Ability to decrease pain	38.7 (20.4)	36.9 (19.3)	32.3 (23.4)

n = number of patients; SD = standard deviation; SF-36 = Medical Outcome Study Short-Form 36; HADS = Hospital Anxiety and Depression Scale; CSQ = Coping Strategies Questionnaire

### Differential score changes within the Multidimensional Pain Inventory subgroups

Table 4 shows entry-to-discharge scores of the three Multidimensional Pain Inventory subgroups. According our main hypothesis, significant differences in pairwise comparison of score changes were found. Comparing the dysfunctional and adaptive copers subgroups, the dysfunctional subgroup showed significantly higher score changes on Multidimensional Pain Inventory life control, Multidimensional Pain Inventory affective distress, and Short Form-36 mental health. The adaptive copers subgroup showed most and significantly higher improvements in Multidimensional Pain Inventory pain severity, Short Form-36 bodily pain, and Coping Strategies Questionnaire ability to decrease pain when compared to the other two subgroups. After Bonferroni correction, the score change of the adaptive copers remained statistically significantly higher than that of the interpersonally distressed in the domain of the four coping dimensions (p = 0.003 < 0.008). In the domain of the four mental (affective) health scales, the score change of the dysfunctional cluster remained statistically significantly higher than those of the interpersonally distressed and the adaptive copers (p = 0.001 < 0.008).

**Table 4:** Cluster score changes and pairwise analyses for significance after the four week pain management program

MPI subgroup	Interpersonally distressed (n = 61)			Adaptive copers (n = 56)			Dysfunctional (n = 50)			p		
**Subscales**	**Entry (m,s)**	**Discharge (m,s)**	**ES (95% CI)**	**Entry (m,s)**	**Discharge (m,s)**	**ES (95% CI)**	**Entry (m,s)**	**Discharge (m,s)**	**ES (95% CI)**	**ID-AC**	**ID-DYS**	**AC-DYS**

**MPI**												
Pain severity	70.4 (16.4)	61.7 (20.3)	**0.53 **(0.24-0.81)	77.3 (13.2)	61.2 (18.1)	**1.22 **(0.86-1.58)	84.3 (11.9)	74.3 (19.3)	**0.84 **(0.44-1.24)	0.006	0.900	0.055
Interference with pain	64.9 (14.1)	57.8 (17.8)	**0.50 **(0.24-0.77)	66.2 (14.7)	54.8 (17.7)	**0.78 **(0.50-1.05)	85.3 (10.2)	76.0 (17.2)	**0.92 **(0.49-1.34)	0.201	0.271	0.847
Life control	48.1 (18.1)	57.2 (20.0)	**0.50 **(0.22-0.79)	63.4 (13.6)	67.5 (16.9)	**0.30 **(-0.06-0.66)	31.9 (17.3)	50.9 (18.2)	**1.10 **(0.76-1.43)	0.289	0.032	0.008
Affective distress	55.7 (21.9)	46.9 (20.4)	**0.41 **(0.20-0.61)	39.7 (18.5)	32.9 (20.2)	**0.37 **(0.11-0.62)	76.1 (16.0)	55.3 (21.7)	**1.30 **(0.91-1.70)	0.650	0.001	0.001
Support	41.2 (24.1)	45.6 (26.1)	**0.18 **(-0.03-0.40)	89.1 (12.0)	83.4 (15.7)	**-0.47 **(-0.74 - -0.21)	87.1 (14.7)	81.6 (17.9)	**-0.38 **(-0.69 - -0.07)	0.001	0.019	0.565
Solicitous responses	32.5 (17.5)	37.9 (23.9)	**0.31 **(0.02-0.60)	77.7 (16.3)	72.3 (20.8)	**-0.33 **(-0.57 - -0.09)	73.6 (17.7)	71.0 (22.7)	**-0.15 **(-0.38-0.09)	0.002	0.017	0.397
General activities	40.8 (12.5)	40.8 (15.4)	**0.00 **(-0.22-0.23)	41.5 (12.9)	42.8 (15.5)	**0.10 **(-0.07-0.27)	32.0 (14.4)	34.9 (17.0)	**0.20 **(-0.01-0.41)	0.691	0.594	0.343

**SF-36**												
Physical functioning	46.0 (20.7)	51.1 (21.4)	**0.25 **(0.05-0.44)	43.1 (22.2)	51.3 (22.6)	**0.37 **(0.16-0.58)	34.7 (19.6)	41.0 (22.0)	**0.32 **(0.12-0.53)	0.675	0.354	0.839
Bodily pain	23.5 (12.7)	31.0 (15.4)	**0.60 **(0.26-0.94)	19.5 (11.1)	30.5 (15.4)	**0.99 **(0.61-1.37)	14.0 (12.8)	18.1 (14.1)	**0.32 **(0.09-0.56)	0.083	0.330	0.007
Mental health	46.8 (20.4)	54.3 (19.1)	**0.37 **(0.16-0.57)	57.8 (19.1)	64.4 (17.5)	**0.34 **(0.15-0.54)	33.4 (13.9)	45.4 (19.1)	**0.86 **(0.55-1.17)	0.926	0.073	0.057

**HADS**												
Anxiety	52.5 (22.1)	57.9 (18.3)	**0.25 **(0.06-0.43)	60.5 (18.5)	66.1 (17.0)	**0.30 **(0.12-0.48)	37.5 (15.4)	43.7 (18.8)	**0.40 **(0.15-0.65)	0.438	0.341	0.585
Depression	57.5 (20.1)	64.6 (21.0)	**0.35 **(0.18-0.53)	65.1 (18.4)	72.9 (20.1)	**0.42 **(0.24-0.60)	39.9 (18.3)	48.0 (24.0)	**0.44 **(0.18-0.71)	0.311	0.655	0.864

**CSQ**												
Catastrophizing	50.2 (17.9)	53.8 (19.4)	**0.20 **(0.02-0.39)	51.2 (15.4)	57.5 (18.8)	**0.41 **(0.19-0.64)	35.1 (17.2)	42.2 (21.2)	**0.41 **(0.17-0.65)	0.141	0.277	0.822
Increasing activity level	55.6 (15.0)	57.2 (17.4)	**0.11 **(-0.13-0.34)	63.8 (13.4)	64.2 (13.7)	**0.03 **(-0.20-0.26)	51.5 (16.9)	54.4 (17.2)	**0.17 **(-0.07-0.41)	0.621	0.766	0.852
Ability to control pain	48.0 (24.4)	48.0 (21.0)	**0.00 **(-0.28-0.28)	48.5 (22.1)	56.5 (18.7)	**0.36 **(0.13-0.60)	33.3 (22.8)	41.5 (22.3)	**0.36 **(0.09-0.63)	0.048	0.009	0.600
Ability to decrease pain	38.7 (20.4)	39.5 (20.0)	**0.04 **(-0.23-0.32)	36.9 (19.3)	49.4 (19.3)	**0.65 **(0.36-0.94)	32.3 (23.4)	38.0 (21.0)	**0.24 **(0.00-0.48)	0.003	0.776	0.025

MPI = Multidimensional Pain Inventory; SF-36 = Medical Outcomes Studies Short Form-36; HADS = Hospital Anxiety and Depression Scale; CSQ = Coping Strategies Questionnaire; m = mean subscale score; s = standard deviation; ES = effect size; p = significance level (type I error) of the Mann-Whitney-U-test

In all three Multidimensional Pain Inventory subgroups the pain management program led to small improvements in physical function and general activities but did not reach significance between subgroups.

## Discussion

According to the main aim of the study, our hypothesis was that the three Multidimensional Pain Inventory subgroups showed significant differences in the change of pain, physical and mental function and coping after the pain management program. This was partly confirmed by our results: The hypothesis was true for the two scales measuring pain reduction - Multidimensional Pain Inventory pain severity and Short Form-36 bodily pain showed highest effects in the adaptive copers subgroup -, three mental health dimensions - Multidimensional Pain Inventory life control, Multidimensional Pain Inventory affective distress, and Short Form-36 mental health showed highest effects in the dysfunctional subgroup -, and the two pain coping dimensions - Coping Strategies Questionnaire ability to control pain and Coping Strategies Questionnaire ability to decrease pain scored in favor of the adaptive coper and dysfunctional subgroups. In our previous study with one hundred and eighteen persons with fibromyalgia, significant differences in treatment outcome across the three Multidimensional Pain Inventory subgroups were mainly observed in favour of the dysfunctional subgroup [23]. Significant subgroup differences in score changes concerning physical functioning and general activities could not be identified.

Talo et al. first showed that different Multidimensional Pain Inventory subgroups might have different treatment outcomes [40]. Patients with poor functional profiles (the subgroups of dysfunctional and interpersonally distressed) may gain a lot from rehabilitation. Three other studies with chronic back pain patients treated in rehabilitation programs did not find inter-group differences [6,7,41]. It was hypothesised that the treatments may not have been relevant to the dysfunctional patients and may not have been sufficiently potent for the interpersonally distressed and adaptive copers patients [28]. In their Multidimensional Pain Inventory subgroup analysis, Vollenbroek-Hutten et al. were able to demonstrate more improvement in the dysfunctional and interpersonally distressed patients, and no differences in the adaptive copers patients [42]. They postulated that the adaptive copers patient group was probably "too good" for an extensive, multidisciplinary, physically oriented rehabilitation program or usual care outside the rehabilitation center. However, the Multidimensional Pain Inventory subgroups were too small to perform a statistical analysis (dysfunctional: n = 9; interpersonally distressed: n = 9; adaptive copers: n = 11). In contrast, the results of the present study showed significant improvements in the different domains of pain reduction, improvement of mental health and pain coping for all three Multidimensional Pain Inventory subgroups, including the adaptive copers patients.

In support of the other hypothesis (secondary aims), the score differences at entry between the subgroups as described by the Multidimensional Pain Inventory were consistent with comparable constructs measured on the Short Form-36, Hospital Anxiety and Depression Scale, and Coping Strategies Questionnaire. The persons in the adaptive copers subgroup showed better general and mental health, less anxiety and depression, less catastrophizing, and better self-efficacy (ability to control and decrease their pain) than persons in the dysfunctional subgroup. The persons in the dysfunctional cluster reported higher levels of pain, anxiety, depression, and use of maladaptive coping strategies, and lowest levels of physical function, social function, and mental health as compared to the adaptive copers subgroup. The interpersonally distressed pattern of the Multidimensional Pain Inventory could not be replicated by the other instruments due to lack of scales with comparable constructs. Overall, the differences of scores between the three clusters implied support for moderate convergent validity.

This retrospective Multidimensional Pain Inventory subgroup classification (*a posteriori*) provides information that may help to improve the effects of standard pain management programs. It suggests matching persons with the treatment strategies and therapeutic methods they are most likely to respond to and where they show the greatest need of treatment and/or where they show the largest deficits (i.e. tailored pain management). Several recent studies in the field of (sub-) acute non specific low back pain and acute neck pain have provided preliminary evidence that using specific inclusion criteria to identify more homogenous subgroups of subjects and attempting to match treatment to the subgroup has the potential to enhance treatment effects [8,12-16]. The development and *a priori *implementation of classification methods for matching interventions of pain management programs to subgroups of patients may improve clinical outcomes. This is being examined in our ongoing randomized controlled trial "Effectiveness of tailored pain management in patients with chronic back pain" (http://www.controlled-trials.com/ ISRCTN25592008/).

In this study, the sample consisted of selected persons who had suffered from a severe and disabling chronic back pain disorder for a long time and who fulfilled certain criteria (e.g. motivation, ability to understand German) and, therefore, possibly differ in important ways from people with chronic back pain in general thus limiting the generalizability of the results. As the design of the study did not include a control group, the changes after the pain program cannot be solely attributed to the interventions. In this singe arm trial it is unclear if the different outcomes for the three subgroups represent different prognosis, response to generic aspects of care, or other factors. Another limitation is the short-term measurement of score changes (between entry and discharge from the rehabilitation clinic after four weeks). Our future study uses standardized outcome measurements not only at the end of the experimental program but at follow-up times that are long enough for the person with chronic back pain to modify behavior patterns and master effective strategies. When using a post-/pre-test difference to define improvement after an intervention, the possibility of regression towards the mean cannot be excluded. We aimed to minimize this effect by using only reliable measurement instruments that have been used previously with persons with chronic back pain. Regression to the mean was not the case in Multidimensional Pain Inventory pain severity, Short Form-36 bodily pain, and Coping Strategies Questionnaire ability to decrease pain. In these three subscales the baseline scores of the dysfunctional subgroups were, compared to the adaptive copers and interpersonally distressed patients, the lowest. However, largest effect sizes in these three subscales were not measured in the dysfunctional subgroup but in the adaptive copers. Finally, all outcome variables were obtained from self-reported questionnaires, e.g. Multidimensional Pain Inventory general activities screening for perceived disability and not from observing physical performance.

## Conclusions

The findings of this study showed that the Multidimensional Pain Inventory subgroups previously identified in less disabled samples of chronic back pain patients are also evident in a highly disabled chronic back pain sample. The three subgroups identified (dysfunctional, interpersonally distressed, and adaptive copers) showed significant differences in score changes in pain, mental health, and coping outcomes following a four week, standard, inpatient, interdisciplinary, pain management program. However, the pain management program led to small but not significant improvements in physical function and general activities.

## Competing interests

The authors declare that they have no competing interests.

## Authors' contributions

MLV, FA, JBS, AA and RAB were responsible for the design of the study. AA procured funding. SL, RB and MLV collected the data. Statistical analysis was performed by FA and MLV. MLV and FA interpreted the data and made a first draft of the manuscript. All authors have read and approved the final manuscript.

## Pre-publication history

The pre-publication history for this paper can be accessed here:

http://www.biomedcentral.com/1471-2474/12/145/prepub
